# Proteolysis modification targeting protein corona affects ultrasound-induced membrane homeostasis of saccharomyces cerevisiae: Analysis of lipid relative contributions on membrane properties

**DOI:** 10.3389/fmicb.2023.1082666

**Published:** 2023-01-26

**Authors:** Zi-Yi Zheng, Guo Xie, Gui-Liang Tan, Wen-Li Liu

**Affiliations:** School of Material Science and Food Engineering, Zhongshan Institute, University of Electronic Science and Technology of China, Zhongshan, China

**Keywords:** protein corona, ferment, nanoparticle, ultrasound, plasma membrane

## Abstract

**Introduction:**

Protein corona (PCN) adsorbed on the surface of nanoparticles has brought new research perspectives for the interaction between nanoparticles and microorganisms. In this study, the responses of *saccharomyces cerevisiae*’ membrane lipid composition, the average length of the fatty acyl chains and the average number of unsaturation of fatty acids to ultrasound combined with nano-Fe_3_O_4_@PCN with time-limited proteolysis (nano-Fe_3_O_4_@TLP-PCN) was investigated.

**Methods:**

Lipidomic data was obtained using Ultra-high performance liquid chromatography coupled with a Q-Exactive plus mass spectrometer. The membrane potential, proton motive force assay and the membrane lipid oxidation were measured using Di-BAC_4_(3), DISC_3_(5) and C11-BODIPY^581/591^ as the probes. Combined with the approach of feasible virtual samples generation, the back propagation artificial neural network (BP-ANN) model was adopted to establish the mapping relationship between lipids and membrane properties.

**Results:**

The time-limited proteolysis targeting wheat PCN-coated Fe_3_O_4_ nanoparticles resulted in regular changes of hydrodynamic diameters, ζ-potentials, and surface hydrophobicity. In addition, with the prolongation of PCN proteolysis time, disturbances of 3 S.cerevisiae membrane characteristics, and membrane lipidomic remodeling in response to ultrasound+ nano-Fe_3_O_4_@PCN were observed. The analysis of relative importance which followed revealed that ergosterol, phosphatidylserine, and phosphatidylinositol phosphate had the greatest influence on membrane potential. For membrane lipid oxidation, ceramide, phosphatidylethanolamine, and sitosterol ester contribute 16.2, 14.9, and 13.1%, respectively. The relative contributions of six lysolecithins to the dissipation of proton motive force remained limited.

**Discussion:**

An adaptation mechanism of cell membrane to proteolyzed PCN, wherein lipidome remodeling could preserved functional membrane phenotypes was revealed. Furthermore, it is highlighted that the relative importances of SiE, Cer, PE and PIP in determining membrane potential, PMF dissipation and membrane lipid oxidation by establishing FVSG-BP-ANN model.

## Introduction

Nowadays, ultrasound (US) is emerging as a useful tool in fermentation applications. Ultrasound-guided shockwaves could alter the liquidity of the phospholipid bilayer of fermentation bacteria, and create nonlethal transient sonopores on fermentation bacteria’s cytomembrane, which might facilitate membrane permeability and release of intracellular enzymes through the cell membrane (Chandan et al., 2020; Choi et al., 2020). For fermentation bacteria, flexible lipidomic remodeling is essential to maintain the physical and chemical properties of the membrane within the range compatible with the life of unicellular organisms (Levental et al., 2020). Interestingly, recent studies revealed that the combination of biocompatible nanoparticles and low-intensity US treatment has shown a satisfactory synergistic effect on the vitality of fermentation bacteria and fermentation efficiency (Li et al., 2020; Zheng et al., 2020). Nanoparticles, as nucleation sites for US-triggered microbubbles, could be directly deposited on a cell (mechanism of sonoprinting). The microbubbles and the rigid surface of the nearby nanoparticles collided with the cell membrane, resulting in stronger dynamic effects of the stretch-compression scattering, reflection wave jet (water hammer), and wicket wave (Blum et al., 2019).

In a fermentation environment, nanoparticles with ultrahigh specific surface area easily interact with surrounding proteins and form a protein corona (PCN), and its surface properties determine the real biological effect of nanoparticles (Lesniak et al., 2013). In most cases, the PCN could reduce cellular internalization and mitigate the cytotoxicity of nanoparticles by preventing interaction between cell membranes and nanoparticles (Lesniak et al., 2013). However, the proteolysis effects of proteases from fermentation microorganisms on PCN and the resultant synergistic effect of US+ nanopartice@PCN on cell membranes have not been studied. Protease might degrade PCN through specific cleavage towards peptide bonds, thus affecting the topological defect of the surface structure, viscoelasticity, and the surface hydrophobic properties (water contact angle) of nanoparticle@PCN. These changes have a profound impact on the dynamic characteristics of microbubbles and the interface force at protein residues/cytoderm (or cytomembrane) contact regions *via* electrostatic force, van der Waals force, hydrophobic force, etc. As mentioned earlier, perturbation of membrane physical properties led to the lipidomic remodeling of the membrane, which was vital for bacteria viability.

Wheat protein is common and inexpensive in the food industry, and it is subdivided into albumin (water-soluble), globulin (salt-soluble), gliadin (alcohol-soluble), and glutenin (residual proteins; Wang et al., 2019). It is worth noting that wheat glutenin which accounts for around 45% of the total wheat protein, is a highly hydrophobic heterogeneous mixture consisting of various subunits connected by disulfide bonds. Therefore, hydrophobic wheat proteins, similar to conventional hydrophobic surfactants, are excellent stabilizing agents for nanoparticles (Mandial et al., 2019).

In this study, the responses of *saccharomyces cerevisiae*’ membrane lipid composition, the average length of the fatty acyl chains, and the average number of unsaturation of fatty acids (FA) to the US combined with nano-Fe_3_O_4_@PCN with time-limited proteolysis (nano-Fe_3_O_4_@TLP-PCN) was investigated. Alterations of 3 membrane properties, including membrane potential, membrane lipid peroxidation, and proton motive force, were also analyzed. Furthermore, BP-ANN models and the accompanying relative importance analysis were implemented to gain in-depth insights into the mapping relationship between membrane lipid composition and membrane properties. In order to solve the problem of the small sample in omics modeling, the data dimension reduction method and virtual sample generation method were conducted and compared.

## Materials and methods

### Materials

The wheat flour was purchased from Yihai Kerry Food Industry Co, LTD (Dongguan, China). Sodium selenite was purchased from Sinopharm Chemical Reagent Co. Ltd. (Shanghai, China). Ultra-pure water was produced by the Milli-Q water purification system (Milford, MA, United States). Bis-(1,3-dibutybarbituric acid) trimethine oxonol (Di-BAC_4_(3)), 3,3′-dipropylthiadicarbocyanine iodide (DISC_3_(5)) and BODIPY^581/591^ C_11_ were purchased from Thermo Fisher Scientific (United States).

### Preparation of cell-free protease extract

The *S. cerevisiae* strain was seeded on yeast extract peptone dextrose (YPD) liquid medium and incubated at 32°C and 150 rpm for 12 h (OD_600_ = 1.2). Then, the cells were harvested using centrifugation (4,000 rpm, 10 min, 4°C). The supernatant was filtered by 0.22 μm membrane and CFPE was obtained.

Sigma’s non-specific protease activity assay was conducted using casein as a substrate. One unit (U/mg) of protease activity of CFPE was defined as the amount of enzyme that is capable of releasing one μmol of tyrosine ml^−1^ per minute (Hui et al., 2019).

### CFPE proteolysis of nano-Fe_3_O_4_@PCN

Fe_3_O_4_ nanoparticles were synthesized according to a previous study (Zheng et al., 2020). Wheat proteins were extracted by *Osborne* extraction procedure, and the obtained wheat proteins solution was incubated with 0.3 mg/ml of prepared nano-Fe_3_O_4_ at room temperature for 1 h under gentle agitation. The obtained nano-Fe_3_O_4_@PCN (Fe concentration of 0.3 mg/ml) were incubated in 5 ml of CFPE (protease activity of 217.3 u/ml) under stirring slowly at 30°C. The proteolysis time was between 0 and 12 h.

### Measurement of nano-Fe_3_O_4_@PCN diameter, ζ-potential, degree of hydrolysis, and surface hydrophobiciy

Particle size distribution and ζ-potential of nano-Fe_3_O_4_@PCN were determined by dynamic light scattering (DLS) measurements (Minić et al., 2022). The OPA (*ortho*-phthalaldehyde) method with some modifications was used to determine the degree of hydrolysis (DH) of protein corona (Moaveni et al., 2022). A total of 1 ml of 0.1 M sodium tetraborate decahydrate solution containing 0.02 mM SDS, 2 μl of β-mercaptoethanol, 20 μl of methanol-OPA (1:200, w/v), and 50 μl of proteolytic protein corona solution or CFPE from *S. cerevisiae* were mixed. Absorbance at 340 nm was measured after 2 min. Glycine was used as standard. DH values were calculated using the following formula:


$$DH\%=\frac{NH_{2ti}-NH_{2t0}-NH_{2ti.CFPE}}{NH_{2Total}-NH_{2ti.CFPE}}\times 100$$

NH_2*ti*_ and NH_2*t*0_ were the free amino groups at i and 0 h. NH_2ti.CFPE_ was the free amino group in the CFPE solution at i h. NH_2Total_ was the free amino group from the whole protein corona.

Octanol–water partition coefficients (K_OW_) of nano-Fe_3_O_4_@PCN were adopted to evaluate the surface hydrophobicity. Equal volumes of Octanol and ultrapure water were combined, and the equilibration process started with the addition of nano-Fe_3_O_4_@PCN (1,10,000, w/v). After 24 h, the mixture was shaken at 50 rpm for 4 h, followed by static separation for 5 h. Nano-Fe_3_O_4_@PCN was collected from each phase, and the concentration of nano-Fe_3_O_4_@PCN was quantified by Inductively Coupled Plasma-optical emission spectroscopy (ICP-OES).

### Combined treatment of US and nano-Fe_3_O_4_@TLP-PCN on *Saccharomyces cerevisiae*

2 Log CFU (colony forming unit) /ml of *S. cerevisiae* were treated by the CFPE-treated nano-Fe_3_O_4_@TLP- PCN (Fe concentration of 0.1 mg/ml) and 45 kHz ultrasonic waves from an ultrasonic bath. The amplitude was 40% (0.0223 W/ml) for 15 s with continuous mode. The power of the US was calculated by the calorimetric method (Peng et al., 2020). The parameter values of US and nano-Fe_3_O_4_@TLP- PCN were chosen according to previous experiments, in order to control the death percentage of *S. cerevisiae* caused by increased membrane permeability (data not shown).

### Lipidomics analysis of the plasma membrane

*Saccharomyces cerevisiae* cells treated by US + nano-Fe_3_O_4_@TLP- PCN were converted to spheroplasts by treatment with EDTA and lysozyme and then disintegrated under sonication (5 min, 0°C) combined with acid-washed glassbeads. The unbroken cells and glassbeads were removed by centrifugation (20,000 *g*, 4°C, 15 min). The resultant cell lysates were ultra-centrifuged (100,000 *g*, 4°C, 120 min). The crude membrane fraction (a mixture of plasma membranes and inner membranes) was collected and subjected to sucrose density gradient centrifugation in MES-buffer (38, 43, and 53% sucrose). After ultracentrifugation for 3 h (100,000 *g*, 4°C), the plasma membrane fraction was collected at 43/53% interface, and then diluted in Tris buffer (20 mM, pH 7.5) and sedimented (80,000 *g*, 4°C, 3 h). The obtained plasma membranes were lyophilized and stored at −80°C.

Lipidomic data was obtained using Ultra-high performance liquid chromatography (UHPLC, Shimadzu, Kyoto, Japan) coupled with a Q-Exactive plus mass spectrometer (Thermo Scientific, Waltham, United States). The extracted membrane lipids were resuspended in acetonitrile/isopropanol (1:9, v/v). The lipids were separated using gradient elution mode (Yuan et al., 2021). The mobile phase consisted of A (6:4 acetonitrile/water with 10 mM ammonium formate) and B (1:9 acetonitrile/isopropanol with 10 mM ammonium formate) under 30% B at 0–2 min, 30–100% B at 3–25 min, 30% B at 25–35 min. The gradient elution modes were identical for positive and negative ESI modes. The MS/MS data were acquired in data-dependent acquisition mode. The full scan spectra covered 200–1800 for positive and negative ESI modes. The resolutions of the full scan (MS^1^) and fragment spectra (MS^2^) were 70,000 and 17,500, respectively. The automatic gain control target values of MS^1^ and MS^2^ were 2 × 10^6^ and 1 × 10^5^. The maximum inject time was 150 ms for MS^1^ and 80 ms for MS^2^. Lipid analysis and identifications were conducted using the LipidSearch (4.1) database.

For ergosterol analysis, an atmospheric-pressure chemical ionization (APCI) source operating in positive-ion detection mode was conducted, and the MS setting was in line with a previous study (Henderson et al., 2013).

The following parameters were used for lipid identification and peak extraction: the quality deviation of precursor ion and product ion in the library was 5 ppm, the response threshold was set as the relative response deviation of product ion (5.0%), the quantitative parameter was set to calculate the peak areas of all identified lipids, and the mass deviation of peak extraction was set to 5 ppm; adduct forms of positive ion mode were [M + H]^+^, [M + NH4]^+^, [M + Na]^+^, and negative ion mode was [M−H]^−^ and [M−2H]^−^ and [M−HCOO]^−^. The original data exported was imported into metaX for data preprocessing, including deleting the lipid molecules missing more than 50% of QC samples, filling the missing value based on the *K*-nearest Neighbor algorithm, and normalizing data using Probabilistic Istic Quotient Normalization.

Each lipid was normalized by the internal standard or the average response of all the used internal standards if no internal standard was available. The average length (*L*) of the fatty acyl chains and the average number (*N*) of unsaturations were calculated as follows:


$$L=\frac{\sum_{k}^{K}n_{k}c_{k}cl_{k}}{\sum_{k}^{K}n_{k}c_{k}}$$

$$N=\frac{\sum_{k}^{K}n_{k}c_{k}i_{k}}{\sum_{k}^{K}n_{k}c_{k}}$$

*n_k_* is the number of fatty acyl chains of each lipid (*k*), *c_k_* is the relative concentration, *cl_k_* is the average carbon length of fatty acyl groups inlipids and *i_k_* is the number of unsaturations.

### Measurement of the membrane potential

2 μg/ml Di-BAC_4_(3) was used to stain the treated *S. cerevisiae* (OD_600_ = 0.5) in black non-transparent 96-well plates at 30°C for 45 min. Membrane potential was detected using flow cytometry (excitation wavelength of 488 nm, emission wavelength of 530 nm).

### Proton motive force assay

The treated cells (OD_600_ = 0.3) were incubated with 0.5 μM DISC_3_(5) in black non-transparent microtiter plates for 15 min. Measurements of DISC_3_(5) fluorescence were conducted using a fluorimeter (622 nm excitation and 670 nm emission filters).

### Measurement of the membrane lipid oxidation

*Saccharomyces cerevisiae* cells which were suspended in 8 mM citrate buffer (pH = 7) and 8 μM C_11_-BODIPY^581/591^ were incubated for 30 min at 30°C and 120 rpm in the dark. Then the cells were treated by nano-Fe_3_O_4_@TLP- PCN and US. To increase the membrane-solubility of the probe, 5 mg/ml of lysozyme and 0.2 M EDTA were added to the suspension. The data of membrane lipid peroxidation were obtained by fluorescence spectroscopy at 500 nm (excitation wavelength) and 520 nm (emission wavelength).

### Data dimension reduction for membrane lipid data

To solve the problem of small sample modeling, data dimension reduction is a powerful alternative method. Effective dimensionality reduction (DR) techniques include principal component analysis (PCA), Laplacian eigen map, locally linear embedding, and autoencoder (AE). First, PCA which is the most commonly used algorithm and adept at linear dimension reduction was conducted in the present study.

Autoencoder is an effective non-linear dimensionality reduction algorithm, which can perform the nonlinear transformation on the high-dimensional input data, map the original high-dimensional features in unsupervised learning, and ensure the integrity of feature information. In order to assess the effectiveness of DR, a series of AE models were established by using membrane lipid data. To optimize the topological structure of the AE model, the number of hidden layers, the number of neurons in each layer, the activation function and the optimization function were adjusted. The number of input data dimensions was set in the range of 2–20. The optimal topology structure was obtained by comparing the reconstruction error which was expressed using mean squared error (MSE). For each AE, MSE was calculated based on the square of the norm of the difference between the lipid data vector obtained from the decoder part of the AE and the actual lipid data vector from the validation data.

### Generation of feasible virtual samples

Generating feasible virtual samples is another feasible approach to solving the modeling of a small sample problem. The process could be divided into 7 steps. Step 1: an extreme learning machine (ELM) was constructed to capture nonlinear mapping between the original data and the extracted features (10-fold cross-validation). The number of hidden layer nodes of the ELM model was determined based on the RMSE (root mean square error) value using trail-and-error method. Step 2: based on the topological manifold concept, Isometric Feature Mapping (Isomap) was adopted to reduce high dimensional data to two visual dimensional spaces, and discover intuitively the sparse data area. Each data point was connected with the 10 nearest neighbor points. Step 3: adequate feasible virtual samples were generated by the half interpolation method in the information gaps, aiming to supplement the space of the original small sample. Step 4: Using the established ELM models, the generated two-dimensional virtual samples were projected back to the original space. Step 5: the outputs (membrane features) of the virtual samples were obtained by the established ELM model. Step 6: virtual samples were screened based on feasibility using an asymmetric acceptable domain range expansion approach. Step 7: the virtual samples and the original training samples were combined into a new training dataset which was used for modifying and validating the ELM model. The performance of the modified ELM model was improved by adding a different number of virtual samples and was evaluated based on RMSE values.

### BP-ANN modeling

BP-ANN which is based on a back propagation algorithm (BP) and artificial neural network (ANN) is applied in the present study to establish the membrane lipid data and membrane properties mapping relationship. BP-ANN is composed of an input layer, multiple hidden layers, and an output layer connected in series, and each layer contains multiple independent neurons in parallel. Adjustable weights between two neurons were applied to stimulate neurons by the activation function.

To successfully train ANNs, especially to prevent overfitting, the dataset should be sufficiently large. One rule-of-thumb is that the size of the sample should be 10 times more than the number of features. However, the sample size of cell membrane lipidomics data was limited in the present study.

To avoid overfitting, a dropout algorithm was adopted in each training epoch to reduce the interdependence between neurons and realize network sparsity processing by discarding part of hidden layer neurons randomly and temporarily. As a consequence, the overfitting of the BP-ANN model was avoided, and the generalization ability was enhanced. The probability of neuron dropout was set at 10–50%. Moreover, an “early stopping” strategy was also adopted in the training process to avoid overfitting. The training process and the testing of model performance were conducted synchronously. Once the AUC (area under the curve) of the training dataset rose and the AUC of the validation dataset descended, the training process was terminated. To ensure robustness, the optimization of BP-ANN was conducted by 10-fold cross-validation.

Garson’s algorithm was used to evaluate the relative importance of lipid species to the membrane properties, and the normalized significance of each predictor was expressed as percentages (Zheng et al., 2020).

## Results

### Characterization of nano-Fe_3_O_4_@TLP-PCN

**Figure 1:**
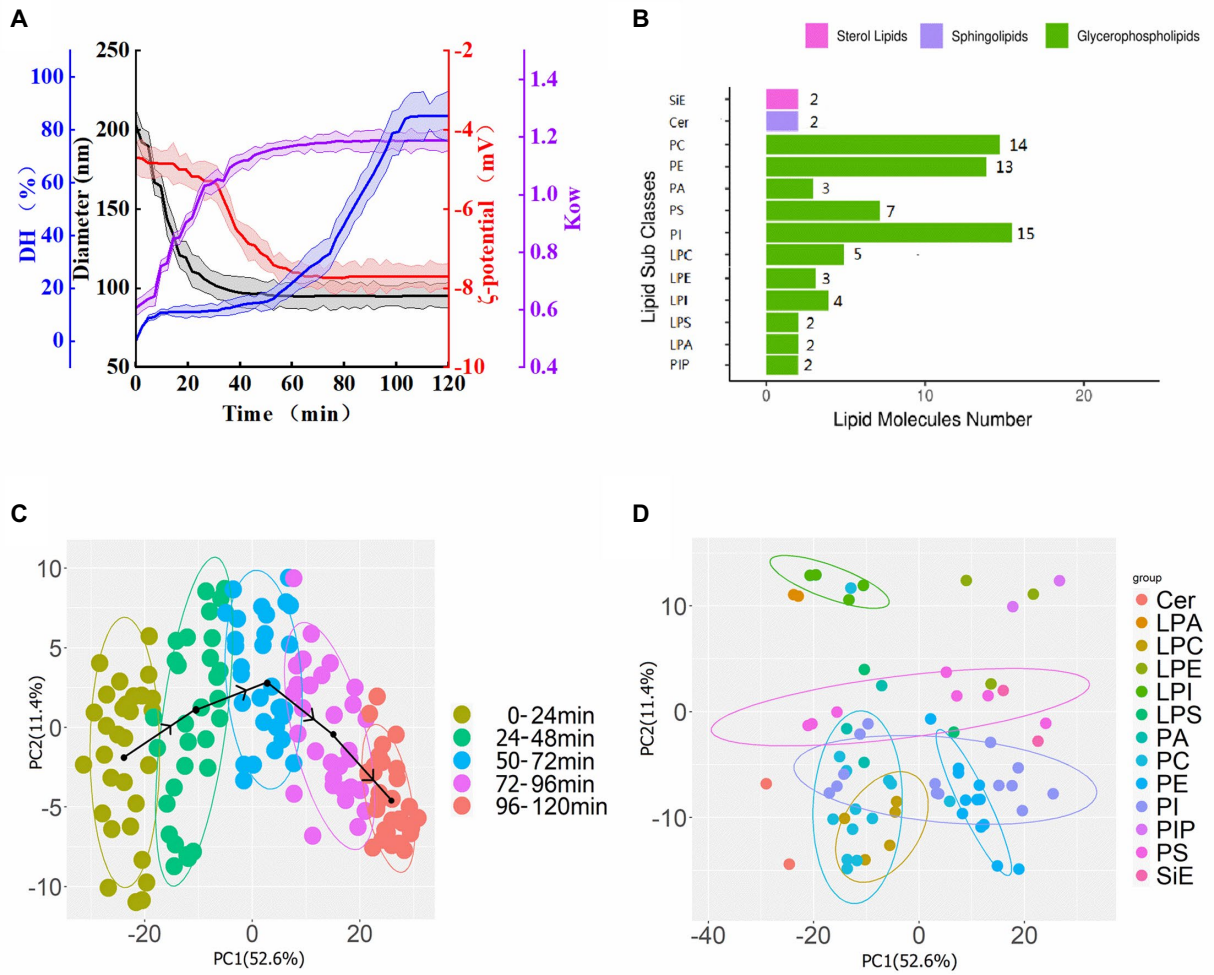
**(A)** Changes of the diameter of nano-Fe_3_O_4_@TLP- PCN (black line), the degree of hydrolysis of protein corona (blue line), ζ-potentials (red line), and surface hydrophobicity (violet line) during PCN proteolysis process. **(B)** 13 membrane lipids subclass and 72 lipid molecular species. **(C)** PCA score plot of membrane lipids colored by samples which were divided into a group every 24 min of proteolysi time. **(D)** PCA loading plot of membrane lipid species.

The results of dynamic light scattering (DLS) revealed that the prepared nano-Fe_3_O_4_ had an average diameter of 71.5 ± 5.4 nm (data not shown). After co-incubation with wheat protein, the diameter of nano-Fe_3_O_4_@ PCN soared to 209 ± 11.6 nm (Figure 1A; Supplementary FigureSupplementary Figure S1). With the extension of proteolysis time, the diameter of nano-Fe_3_O_4_@TLP- PCN gradually dwindled to 92.5 ± 9.8 nm and stopped descending after 60 min.

In consideration of the molecular size of wheat protein, DLS results indicated that the PCN on the surface of nano-Fe_3_O_4_ was a mult-layer PCN. Moreover, the diameter of nano-Fe_3_O_4_@ PCN which underwent full proteolysis was significantly higher than the diameter of original nano-Fe_3_O_4_, indicating that there was an unable-to-be proteolyzed PCN layer on the contact surface of nano-Fe_3_O_4_.

The OPA method with some modifications was used to determine the degree of hydrolysis (DH) of the protein corona，and the CFPE solution was the blank group. It was found that DH values of PCN started at 0% and gradually increased to 85.2 ± 5.1% during 12 h of CFPE-proteolysis, as shown in Figure 1A (blue line). Meanwhile, during the proteolysis of PCN, the average surface charge density (ζ-potential) of nano-Fe_3_O_4_@TLP-PCN was shifted towards lower values (from −4.7 to −7.8 mV; Figure 1A, red line). Specifically, ζ-potential showed a slow descent (0–30 min) followed by a fast descent (30–50 min), and then it ran at a level close to −7.8 mV.

Octanol–water partition coefficient (K_OW_) is defined as the ratio of the concentration of a chemical or nanoparticle in the octanol phase to its concentration in the aqueous phase at equilibrium. When the K_OW_ value is higher than 1, it indicates the hydrophobic property of nanoparticles, and larger values indicate a greater hydrophobic property of nanoparticles. The results showed that the K_OW_ of nano-Fe_3_O_4_@TLP-PCN increased from 0.61 to 1.19 (Figure 1A, violet line), indicating that CFPE digestion might expose more hydrophobic regions of PCN.

### Analysis of membrane lipidomics

It is critical to understand the interaction between nanoparticles and fermentation bacteria from the perspective of membrane lipids, which is helpful for designing more efficient nanoparticles and PCN. The typical total ion chromatogram of membrane lipids extract was shown in Supplementary FigureSupplementary Figure S2. The lipids of unidentified in ESI+ and ESI− modes were discarded, and the list of 72 unique compounds and information (retention time, *m*/*z*, YMDB code, and major adducts) were shown in Supplementary FigureSupplementary Table S1. A total of 13 lipid subclasses were identified in *S. cerevisiae* membranes, including 15 phosphatidylinositols (PI), 14 phosphatidylcholines (PC), 13 phosphatidylethanolamines (PE), 7 phosphatidylserines (PS), 3 phosphatidic acids (PA), 2 ceramides (Cer), ergosterol (Figure 1B). Additionally, phospholipids (62.5–67.1%) predominated in all groups, followed by sphingolipids (12.4–18.2%) and sterol lipids (11.5–17.6%). Most phospholipids contained either 32 or 34 carbons, and most phospholipids contained no more than 2 double bonds. Moreover, only saturated and monounsaturated FA chains were observed, which was in line with a previous study (Danne-Rasche et al., 2020).

Principal component analysis was performed by using lipidomics data from 50 samples (Figure 1C). The score plot exhibited the distribution of the individuals of 50 samples (3 biological replicates) which were treated with different nano-Fe_3_O_4_@TLP-PCN in two-dimensional space, and two principal components captured 64% changes in lipid composition. The black arrow lines connecting the points which were the centers of 5 confidence ellipses showed how the scores evolved with the proteolysis time of PCN. Score plots indicated that membrane lipid profiles in samples treated with PCN_96-120 min_ were negatively correlated with those in the samples treated with PCN_0-24 min_ on the axis of principal component 1. It was worth noting that the black arrow lines presented a reversed “U” shape, demonstrating that some specific lipid species presented call-back tendencies along with the proteolysis of PCN. Additionally, clear discrimination from two samples whose interval of the proteolysis time was higher than 24 min was found. Meanwhile, correlations between lipid species and principal components were found in the loading plot (Figure 1D). A total of 8 of 13 PIs and all PEs presented positive correlations with principal component-1, whereas all PAs, the remaining PIs, all PC and LPC were negatively correlated with principal component 1. Additionally, PC(12:0/18:2), PC(15:0/18:2), PC(16:0/18:1), PA(36:2), and PE(16:0/18:1) gathered in the center of the loading plot, suggesting their unimportant contribution in the sample-grouping.

**Figure 2:**
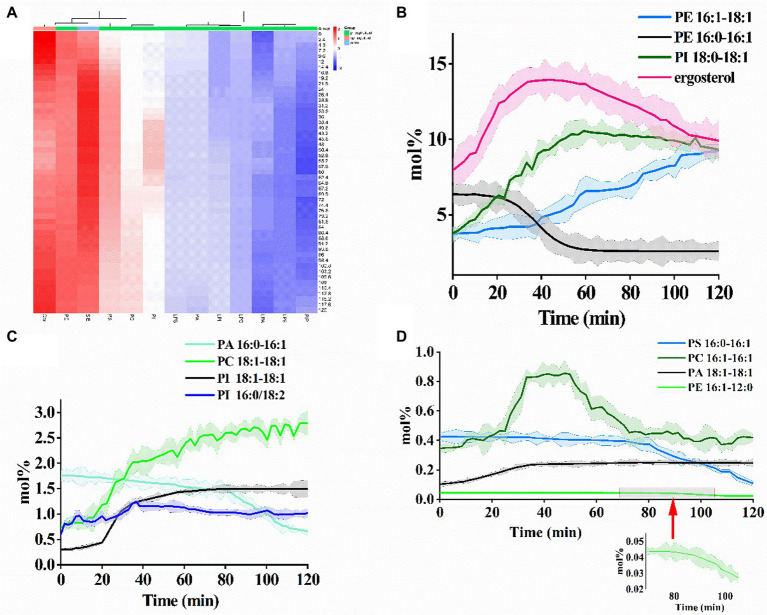
**(A)** Heatmap providing the relative abundance change of each identified lipid in cytomembrane during the PCN proteolysis process. Quantitative analysis of the compositional changes of 12 phospholipids and sterols with **(B)** high-abundance, **(C)** middle-abundance, and **(D)** low-abundance.

Furthermore, alterations in the relative abundances of membrane lipid subclasses with the proteolysis time of PCN were summarized as a heatmap (Figure 2A). Unlike most phospholipids, the relative abundance of sphingolipids first decreased and then increased with the prolongation of proteolysis time. It was observed that the relative abundances of PE maintained stability with the increased proteolysis time, while the relative abundances PS and PI reached their maximum at proteolysis time of 40.8 and 55.2 min, respectively.

The concentration changes of 12 phospholipids and sterols were quantitatively analyzed (Figures 2B–D). The concentrations of the selected lipids ranged from 0.023 mol% (PE 16:1–12:0) to 13.95 mol% (ergosterol). Concentrations of PE (16:1–18:1, high-abundance), PC (18:1–18:1, middle-abundance), PI (18:1–18:1, middle-abundance), and PA (18:1–18:1, low-abundance) elevated and the rising speed slowed down with the increased proteolysis time of PCN. Particularly, PI (18:1–18:1, middle-abundance) showed an S-shaped growth. Conversely, concentrations of PE (16:0–16:1, high-abundance), PA (16:0–16:1, middle-abundance), PS (16:0–16:1, low-abundance), and PE (16:1–12:0, low-abundance) exhibited downward trajectory. PE (16:0–16:1, high-abundance) showed an S-shaped decline. Apart from the abovementioned phospholipids, concentrations of the remaining lipids including ergosterol, PI (18:0–18:1), PI (16:0/18:2), and PC (16:1–16:1) increased followed by a decline with the rising of proteolysis time. Moreover, the levels of all four phospholipids with 2 unsaturated double bonds increased.

**Figure 3:**
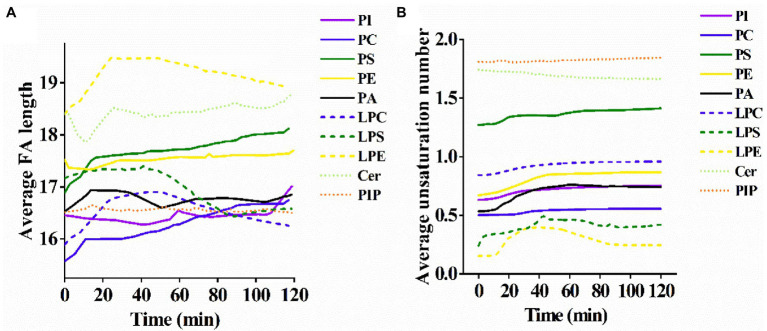
**(A)** Alterations of the average length of the fatty acyl chains and **(B)** the average number of unsaturations in membrane lipids of *S. cerevisiae* exposed to US+ nano-Fe_3_O_4_@TLP- PCN with different proteolysis time.

From the results of the average FA chain length for each lipid subclass, PC, PI, and PS showed a slow rise in the average FA chain length with the increase of PCN proteolysis time, whereas the average FA chain length of Cer, phosphatidylinositol phosphate (PIP) and PA exhibited an ambiguous trend in response to the proteolysis time (Figure 3A). The average FA chain length of PE and PIP remained basically stable.

The alterations of the unsaturation level for each lipid subclass were also investigated. Except for Cer, lysophosphatidylethanolamine (LPE) and lipopolysaccharide (LPS), the average unsaturation level for most lipids rose slowly with the prolongation of proteolysis time (Figure 3B). It was also noticed that the increments of unsaturation levels of PA and PE were higher than those of PI, PS, and PC.

### Changes in plasma membrane properties

**Figure 4:**
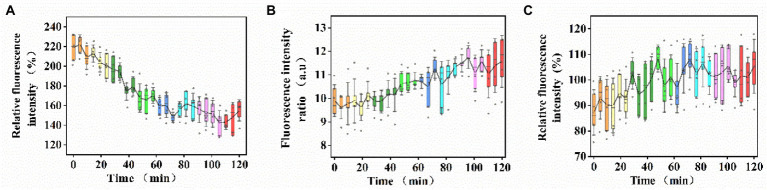
The effects of US+ nano-Fe_3_O_4_@TLP- PCN with 0–120 min of proteolysis on the **(A)** membrane potential, **(B)** membrane lipid oxidation, and **(C)** dissipation of PMF.

In consideration of that self-adaptive remodeling of membrane lipids and recovery of baseline physical properties are temporally regulated, the time point of sample collection must be set as early as possible after US+ nano-Fe_3_O_4_@TLP-PCN treatment. The effect of US+ nano-Fe_3_O_4_@TLP- PCN on the membrane potential of *S. cerevisiae* was investigated by measuring the fluorescence intensity of Di-BAC_4_(3). From Figure 4A, it was found that the membrane potential decreased with the prolongation of PCN proteolysis. Moreover, US+ nano-Fe_3_O_4_@TLP- PCN resulted in an insignificant increase of membrane lipid oxidation and dissipation of proton-motive force (PMF) which was equal to the sum of transmembrane electrical potential (Δ*ψ*) and transmembrane proton gradient (ΔpH; Figures 4B,C).

### BP-ANN model establishment and verification

**Figure 5:**
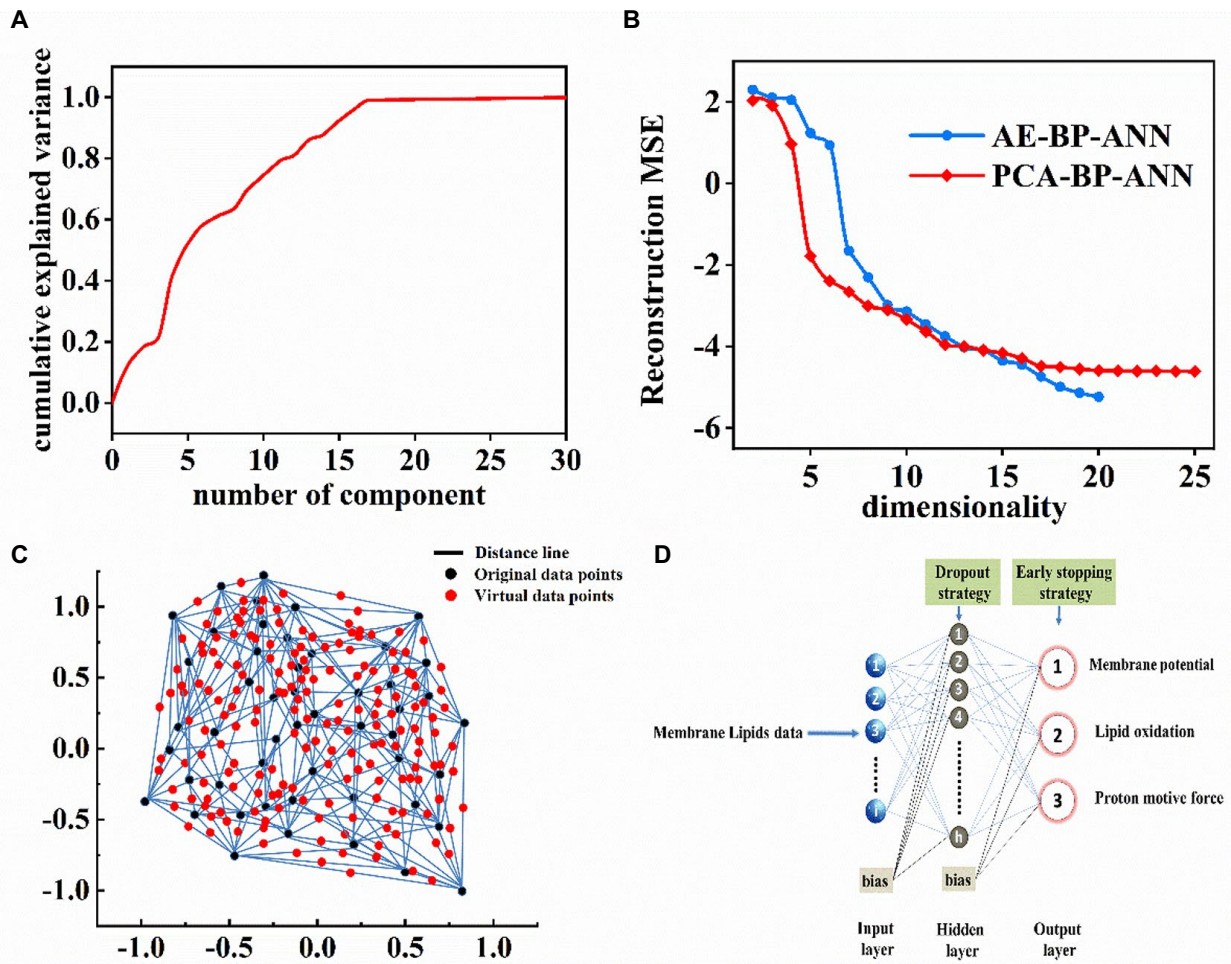
**(A)** The relationship of principal components number and cumulative explained variance (%). **(B)** Reconstruction MSE in the logarithmic scale as a function of the input data dimension number based on PCA-BP-ANN and AE-BP-ANN models. **(C)** Two-dimensional interpolation graph of virtual sample points. **(D)** BP-ANN topology architecture.

Prior to BP-ANN modeling, two approaches, i. e., data dimension reduction and virtual sample generation were taken into consideration to prevent overfitting in small-sample BP-ANN modeling. First, PCA and AE were applied to reduce the dimensionality of membrane lipids data. The results showed that the first 17 principal components could explain more than 99% variance of the original lipid data (Figure 5A). Figure 5B exhibited the calculated MSE as a function of the dimensionality based on the BP-ANN model with principal components as the input dataset. It was clear that the dimension of the input data space could be reduced to 8 with MSE < 10^−3^. Similarly, for dimension reduction of the AE approach, the number of input data dimensions was eventually chosen as 10.

To achieve feasible virtual sample generation (FVSG), Isomap was established to reduce high-dimensional data to two visual dimensional space (Figure 5C; black points) and numerous feasible virtual samples (Figure 5C; red points) were generated by the semi-interpolation method in the information gaps. The average RMSE achieved a minimum of 0.228 when the number of random virtual samples was 227. Therefore, the merged dataset consisted of 227 virtual samples and 50 original samples.

First, 8 principal components and 10 new features obtained by PCA and AE were used as the input data for BP-ANN model training, validation, and test. Considering that the predictive power of BP-ANN hinged on the randomness of training data and validation data, the data consisted of 50 samples that were randomly divided into 60% training dataset, 20% validation dataset, and 20% test dataset.

**Figure 6:**
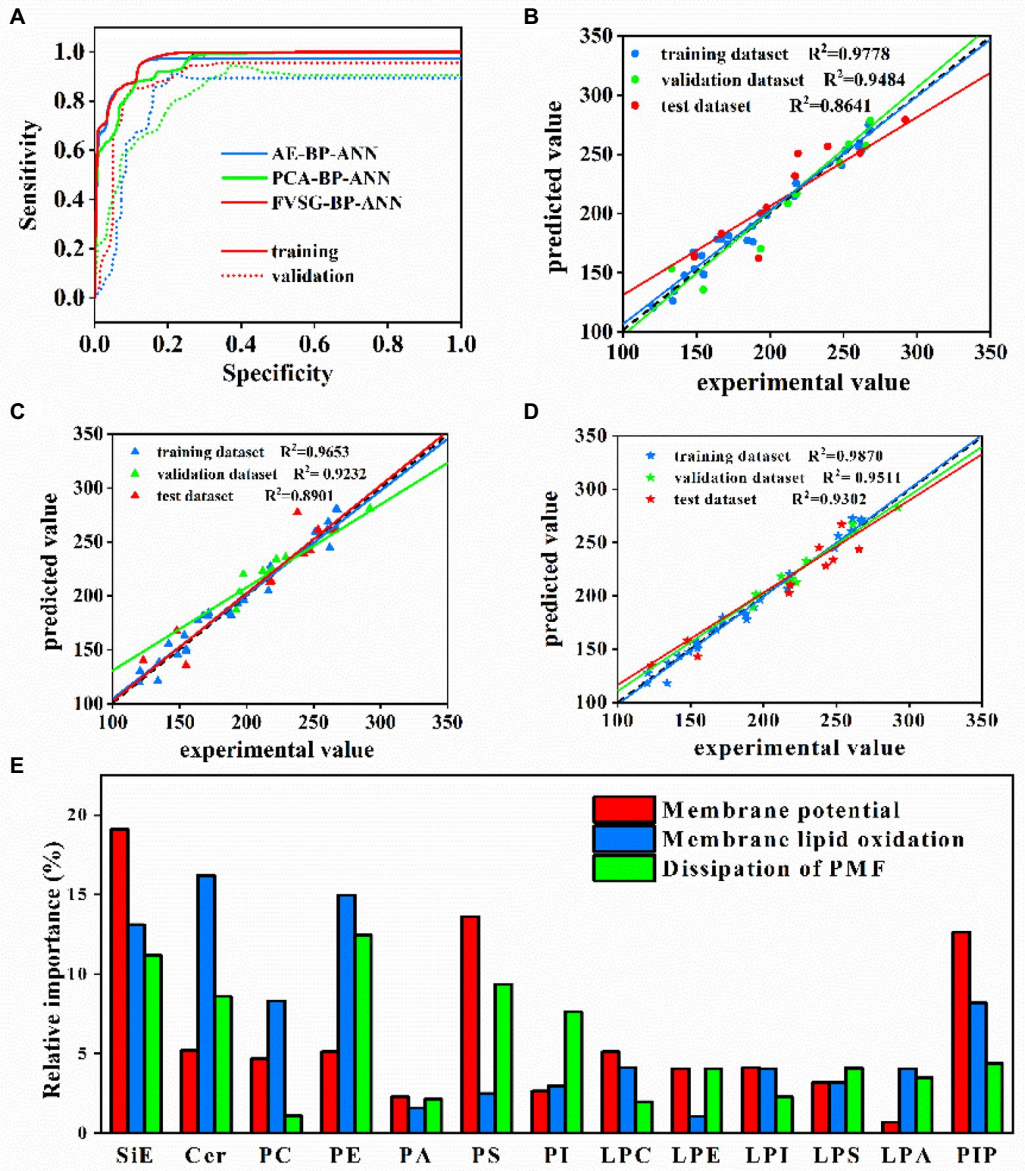
**(A)** Training and validation AUCs of 3 BP-ANN models. **(B)** PCA-BP-ANN, **(C)** AE-BP-ANN, and **(D)** FVSG-BP-ANN predictions of membrane potential vs. experimental values during training, validation, and testing. **(E)** Relative importance analysis (variations of membrane lipids as the input data to membrane properties as the output data).

Second, the strategy of early-stopping was applied to achieve the best results for each case (Figure 5D). For instance, Figure 6A showed that the AUC of AE-BP-ANN on the validation dataset rose during the first 65 epochs, but began to descend after 66 epochs, while AUC on the training dataset gradually increased and then maintained constant eventually before 300 epochs. Similarly, the early-stop strategy was also conducted at 98 epochs for PCA-BP-ANN training. However, there was no decline in the AUC of FVSG-BP-ANN on the validation dataset, and MSE for FVSG-BP-ANN training and validation stopped falling after 342 epochs. Taking the above results into account, it was recommended training should stop at 65,98, and 352 epochs for AE-BP-ANN, PCA-BP-ANN, and FVSG-BP-ANN, respectively.

Third, Figures 6B–D showed the comparison between the predicted values of membrane potential and experimental values in 3 BP-ANN models on training, validation, and test datasets. The predictive performances of PCA-BP-ANN, AE-BP-ANN, and FVSG-BP-ANN models for membrane potential were evaluated based on the regression coefficients (*R*^2^) of the test dataset, which were 0.8641, 0.8901, and 0.9302, respectively. In addition, test dataset *R*^2^ of membrane lipid oxidation and dissipation of PMF were exhibited in Supplementary FigureSupplementary Figure S3. Under the premise of equal weight, the average *R*^2^ of 3 membrane properties in the test dataset was the highest in the FVSG-BP-ANN model (Supplementary FigureSupplementary Figure S3). Consequently, the highest predictive ability of the model was FVSG-BP-ANN.

### Relative importance analysis of lipids to membrane properties

Relative importance analysis (sensitivity analysis) based on the established FVSG-BP-ANN model was applied to calculate the relative importance of each lipid subclass to 3 membrane properties. As shown in Figure 6E, the most important predictor of membrane potential was ergosterol, followed by PS and PIP. Meanwhile, LPA and PA had a slight influence on the membrane potential. For membrane oxidation, Cer, PE, and SiE were identified as the top three important lipids. Furthermore, the normalized relative importance of PE, ergosterol, and PS to PMF dissipation of membrane was higher than 9%.

## Discussion

According to a previous study, PCN may trigger the aggregation of nanoparticles through protein bridges, resulting in a larger apparent hydrodynamic diameter (Dominguez-Medina et al., 2013). In contrast, the existence of a protein corona may also stabilize nanoparticles and prevent aggregation in different conditions. In the present study, the diameter of nano-Fe_3_O_4_@PCN showed a gradual decrease during the process of PCN proteolysis. The cleavage of peptide bonds in PCN during CFPE-hydrolysis disrupted the intact wheat protein structure, and most of the resultant peptide residues on the surface of PCN might be amorphous with different chain lengths (Zhao et al., 2021). Hence, the PCN surface with a “brush-shape” failed to adsorb the surrounding peptide or protein. Moreover, the results of ζ-potential showed that the increased surface electrostatic repulsion might result in decreased aggregation of the nanoparticles. On the other hand, the analysis of the surface hydrophobicity indicated that more hydrophobic regions of PCN were exposed to CFPE digestion. Exposure of the hydrophobic patches on the outer surface of PCN might also help to prevent the attachment of proteins or peptides in the surrounding medium to the outer surface of PCN (Ma et al., 2020).

In addition, proteins adsorbed to nanoparticles’ surfaces might undergo either reversible or irreversible conformational changes, even forming protein aggregates (Lesniak et al., 2013). The changes in protein conformation of PCN could be uncovered using cross-linking mass spectrometry (XL-MS) technique in subsequent studies. The steric hindrance and polymer shielding caused by the interface interaction of nanoparticle-protein might result in resistance of the innermost PCN layer to protease.

The alterations of PCN surface hydrophobicity, ζ-potential, and diameter of nanoparticles during the CFPE proteolysis process might result in regular changes of membrane lipid. In consideration that cell membrane lipids are hydrophobic, it was found that PCN surfaces with higher hydrophobicity might have a greater impact on membrane lipid and membrane properties in the present work. A previous study exhibited that PCN interacted more strongly with the anionic leaflet of the cell membrane than with the zwitterionic leaflet，resulting in significantly disordered anionic leaflets (Lee, 2021). Interestingly, our results also showed the maximum fold-change of anionic PI among all lipid subclasses, and smaller fold-change of neutral lipids including PC and PE. PI and PS have the common substrate cytidine diphosphat-diacylglycerin, indicating that they have a competitive relationship (Liu et al., 2022). However, the change trends of PI and PS are similar in the present study.

To adapt to complex and changeable circumstances, microorganisms could change the membrane properties by adjusting membrane lipids. Membrane lipid composition could be controlled by regulating the type of phospholipid tail group and the distribution of the phospholipid head group (Nasution et al., 2017). The phospholipid tail type could also be altered by adjusting the unsaturation level of lipids and modifying the length of the acyl chains. FAs of 16 or 18 carbon atoms, which serve critical functions including energy source, protein modifiers, and signal molecules, are the preferred acyl substance of eukaryotic membrane lipids (Enkavi et al., 2019). According to the result of previous research, the membrane with a long FA chain length was relatively thick and stable in the fluid phase (Henry et al., 2006). For membrane lipid unsaturation, an increased degree of membrane lipid unsaturation might enhance the steady state of yeast cells in coercion (Jordá et al., 2020). According to the results of FA chain length and unsaturation level, it could mean that PCN with higher DH exerted a greater coercive effect on *S. cerevisiae.* It was also noticed that unsaturation levels of PA and PE rose more than PI, PS, and PC, indicating the priority of unsaturation change in different lipid subclasses.

Although the cell membrane is negatively charged, some patchy areas with cationic sites might allow the binding of the negatively charged PCN, resulting in the disturbance of membrane lipids (Dominguez-Medina et al., 2013). Furthermore, amphiphilic and hydrophobic peptides on the PCN surface were inserted into the *S. cerevisiae* membrane through hydrophobic interaction, resulting in a slight potential change.

The result of membrane lipid oxidation was nearly identical to that of a previous study, which indicated that membranes rich in saturated FAs are less sensitive to lipid oxidation (Rysman et al., 2010). Meanwhile, the altered abundance of lipid rafts might also protect cells from lipid oxidation (Beloribi-Djefaflia et al., 2016).

In previous research, the adaption of membrane homeostasis has been preliminarily deduced from the data-driven model by using physicochemical properties of the lipid matrix, including curvature and melting temperature (Dymond, 2015). Nevertheless, the extrapolation of membrane physical parameters based on the *in vitro* model may not be a reliable approach. In the present work, the BP-ANN model was applied to establish the relationships between membrane lipids and membrane properties.

Negatively charged PS and PI have a considerable impact on the electrostatic properties of the membrane, which is consistent with our findings (Agarwala et al., 2022). As for PI(4)P, it drives the distribution of other membrane lipids and appeared as an important determinant of the whole membrane structure (Banerjee and Kane, 2020). PA is a precursor to other phospholipids, and also a signaling molecule (Walker et al., 2020). On the other hand, a previous study indicated that the membrane homeostasis of prokaryotes is usually regulated by phospholipids, whereas the membrane properties of most eukaryotes are mediated by sterols (Crockett, 1998). The present work also confirmed the importance of ergosterol to membrane potential, PMF dissipation, and membrane lipid oxidation. Severe PMF collapse leads to the loss of bacterial viability and affects cell volume and expansion by driving transport across the cell membrane (Morth et al., 2011; Jia et al., 2022). According to the results of the relative importance analysis, it could be deduced that the change of the Cer/PE/SiE ratio is one of the adaptive mechanisms of *S. cerevisiae* to oxidative stress induced by US+ nanoparticles.

## Conclusion

In cooperation with the US, PCN with different proteolysis degrees strongly impacted membrane lipids compositions, and several lipids exhibited monotonicity change. Fold-change of PS (16:0–16:1), PIP, and PI (18:1–18:1) could be 3.94, 4.53, and 5.04, respectively. In regard to lipid subclasses, PE, PA, and LPS were barely affected by the proteolysis degree of PCN. In view of the results that the variation extents of membrane lipids subclasses were less severe than those of lipid molecular species, it could be inferred that membrane lipidic adaptation to the different PCN might be instead addressed by modifying the proportions of the different lipid molecular species. We found a minor increase in the FA chain length of PI, PC, and PS and a slight increase in the average unsaturation of most lipids with the increase in the degree of PCN proteolysis. The changes in membrane potential, dissipation of PMF, and membrane lipid oxidation were less than 26.7%. These results revealed an adaptation mechanism of the cell membrane to proteolyzed PCN, wherein lipidome remodeling preserved functional membrane phenotypes. Furthermore, we highlighted the relative importance of SiE, Cer, PE, and PIP in determining membrane potential, PMF dissipation, and membrane lipid oxidation by establishing the FVSG-BP-ANN model.

## Data availability statement

The original contributions presented in the study are included in the article/Supplementary FigureSupplementary material, further inquiries can be directed to the corresponding author.

## Author contributions

Z-YZ and W-LL: conceptualization and methodology. GX: software and resources. G-LT and Z-YZ: validation. Z-YZ: formal analysis, investigation, writing—original draft preparation, writing—review and editing, and funding acquisition. W-LL: data curation and visualization. G-LT: supervision and project administration. All authors contributed to the article and approved the submitted version.

## Funding

This study was financed by the National Natural Science Foundation of China (Grant No. 32101903), the Guangdong Basic and Applied Basic Research Foundation (Grant No. 2020A1515011308), and Guangdong Provincial Science and Technology Project— University Science park project (Grant No. 2021A0101180005).

## Conflict of interest

The authors declare that the research was conducted in the absence of any commercial or financial relationships that could be construed as a potential conflict of interest.

## Publisher’s note

All claims expressed in this article are solely those of the authors and do not necessarily represent those of their affiliated organizations, or those of the publisher, the editors and the reviewers. Any product that may be evaluated in this article, or claim that may be made by its manufacturer, is not guaranteed or endorsed by the publisher.
