# Supplementary material for: Proteolysis modification targeting protein corona affects ultrasound-induced membrane homeostasis of saccharomyces cerevisiae: Analysis of lipid relative contributions on membrane properties
Source: Front Microbiol. 2023 Jan 26;14:1082666. doi: 10.3389/fmicb.2023.1082666 (PMC9909265; doi:10.3389/fmicb.2023.1082666)
Supplement: Supplementary file 1 [file Data_Sheet_1.docx]

**TABLE S1**. Lipid assignment from UHPLC-MS data.

| **Measured *m/z*** | **Retention Time** | **LipidIon** | **Lipid Group** | **Adduct** | **Class** | **Fatty Acid** | **FA1** | **FA2** | **FA3** | **Calculated *m/z*** |
| --- | --- | --- | --- | --- | --- | --- | --- | --- | --- | --- |
| 562.51939 | 7.87 | Cer(d36:3) | Cer(d36:3) | M+H | Cer | d36:3 | d36:3 | NA | NA | 562.5194 |
| 588.53561 | 7.91 | Cer(d38:2) | Cer(d38:2) | M+H | Cer | d38:2 | d38:2 | NA | NA | 588.535 |
| 538.31463 | 0.94 | LPC(16:1) | LPC(16:1) | M+HCOO | LPC | 16:01 | 16:01 | NA | NA | 538.315 |
| 568.36232 | 1.61 | LPC(18:0) | LPC(18:0) | M+HCOO | LPC | 18:00 | 18:00 | NA | NA | 568.362 |
| 566.34714 | 1.17 | LPC(18:1) | LPC(18:1) | M+HCOO | LPC | 18:01 | 18:01 | NA | NA | 566.3463 |
| 564.33133 | 1 | LPC(18:2) | LPC(18:2) | M+HCOO | LPC | 18:02 | 18:02 | NA | NA | 564.3307 |
| 550.38784 | 1.68 | LPC(20:1) | LPC(20:1) | M+H | LPC | 20:01 | 20:01 | NA | NA | 550.3867 |
| 409.23623 | 1.18 | LPA(16:0) | LPA(16:0) | M-H | LPA | 16:00 | 16:00 | NA | NA | 409.2361 |
| 437.26740 | 1.62 | LPA(18:0) | LPA(18:0) | M-H | LPA | 18:00 | 18:00 | NA | NA | 437.2674 |
| 454.29306 | 1.23 | LPE(16:0) | LPE(16:0) | M+H | LPE | 16:00 | 16:00 | NA | NA | 454.2928 |
| 452.27706 | 0.98 | LPE(16:1) | LPE(16:1) | M+H | LPE | 16:01 | 16:01 | NA | NA | 452.2772 |
| 482.32450 | 1.71 | LPE(18:0) | LPE(18:0) | M+H | LPE | 18:00 | 18:00 | NA | NA | 482.3241 |
| 571.28939 | 0.9 | LPI(16:0) | LPI(16:0) | M-H | LPI | 16:00 | 16:00 | NA | NA | 571.2889 |
| 569.27378 | 0.74 | LPI(16:1) | LPI(16:1) | M-H | LPI | 16:01 | 16:01 | NA | NA | 569.2732 |
| 599.32060 | 1.18 | LPI(18:0) | LPI(18:0) | M-H | LPI | 18:00 | 18:00 | NA | NA | 599.3202 |
| 597.30490 | 0.89 | LPI(18:1) | LPI(18:1) | M-H | LPI | 18:01 | 18:01 | NA | NA | 597.3045 |
| 496.26801 | 0.92 | LPS(16:0) | LPS(16:0) | M-H | LPS | 16:00 | 16:00 | NA | NA | 496.2681 |
| 522.28350 | 0.91 | LPS(18:1) | LPS(18:1) | M-H | LPS | 18:01 | 18:01 | NA | NA | 522.2837 |
| 645.45122 | 5.09 | PA(16:0/16:1) | PA(32:1) | M-H | PA | 16:0/16:1 | 16:00 | 16:01 | NA | 645.4501 |
| 699.49754 | 6.41 | PA(18:1/18:1) | PA(36:2) | M-H | PA | 18:1/18:1 | 18:01 | 18:01 | NA | 699.497 |
| 718.53822 | 7.74 | PA(36:2) | PA(36:2) | M+NH4 | PA | 18:1/18:1 | 18:01 | 18:01 | NA | 718.5381 |
| 748.51507 | 4.38 | PC(16:1/14:0) | PC(30:1) | M+HCOO | PC | 16:1/14:0 | 16:01 | 14:00 | NA | 748.5134 |
| 746.49814 | 3.61 | PC(12:0/18:2) | PC(30:2) | M+HCOO | PC | 12:0/18:2 | 12:00 | 18:02 | NA | 746.4978 |
| 778.56165 | 6.98 | PC(16:0/16:0) | PC(32:0) | M+HCOO | PC | 16:0/16:0 | 16:00 | 16:00 | NA | 778.5604 |
| 776.54838 | 5.62 | PC(16:0/16:1) | PC(32:1) | M+HCOO | PC | 16:0/16:1 | 16:00 | 16:01 | NA | 776.5447 |
| 774.53231 | 4.52 | PC(16:1/16:1) | PC(32:2) | M+HCOO | PC | 16:1/16:1 | 16:01 | 16:01 | NA | 774.5291 |
| 790.56094 | 6.36 | PC(15:0/18:1) | PC(33:1) | M+HCOO | PC | 15:0/18:1 | 15:00 | 18:01 | NA | 790.5604 |
| 788.54654 | 5.19 | PC(15:0/18:2) | PC(33:2) | M+HCOO | PC | 15:0/18:2 | 15:00 | 18:02 | NA | 788.5447 |
| 804.57916 | 7.19 | PC(16:0/18:1) | PC(34:1) | M+HCOO | PC | 16:0/18:1 | 16:00 | 18:01 | NA | 804.576 |
| 704.52273 | 4.4 | PC(30:1) | PC(30:1) | M+H | PC | 16:1/14:0 | 16:01 | 14:00 | NA | 704.5225 |
| 702.50687 | 3.63 | PC(30:2) | PC(30:2) | M+H | PC | 12:0/18:2 | 12:00 | 18:02 | NA | 702.5068 |
| 734.57003 | 7.03 | PC(32:0) | PC(32:0) | M+H | PC | 16:0/16:0 | 16:00 | 16:00 | NA | 734.5694 |
| 754.53564 | 5.65 | PC(32:1) | PC(32:1) | M+Na | PC | 16:0/16:1 | 16:00 | 16:01 | NA | 754.5357 |
| 730.53910 | 4.56 | PC(32:2) | PC(32:2) | M+H | PC | 16:1/16:1 | 16:01 | 16:01 | NA | 730.5381 |
| 782.56684 | 7.24 | PC(34:1) | PC(34:1) | M+Na | PC | 16:0/18:1 | 16:00 | 18:01 | NA | 782.567 |
| 688.49470 | 6.03 | PE(16:0/16:1) | PE(32:1) | M-H | PE | 16:0/16:1 | 16:00 | 16:01 | NA | 688.4923 |
| 686.47833 | 4.81 | PE(14:0/18:2) | PE(32:2) | M-H | PE | 14:0/18:2 | 14:00 | 18:02 | NA | 686.4766 |
| 714.51044 | 5.99 | PE(16:1/18:1) | PE(34:2) | M-H | PE | 16:1/18:1 | 16:01 | 18:01 | NA | 714.5079 |
| 632.43023 | 3.62 | PE(16:1/12:0) | PE(28:1) | M-H | PE | 16:1/12:0 | 16:01 | 12:00 | NA | 632.4297 |
| 716.52585 | 4.46 | PE(16:0/18:1) | PE(34:1) | M-H | PE | 16:0/18:1 | 16:00 | 18:01 | NA | 716.5236 |
| 714.51015 | 3.79 | PE(16:0/18:2) | PE(34:2) | M-H | PE | 16:0/18:2 | 16:00 | 18:02 | NA | 714.5079 |
| 742.53961 | 4.6 | PE(18:1/18:1) | PE(36:2) | M-H | PE | 18:1/18:1 | 18:01 | 18:01 | NA | 742.5392 |
| 690.50648 | 6.12 | PE(32:1) | PE(32:1) | M+H | PE | 16:0/16:1 | 16:00 | 16:01 | NA | 690.5068 |
| 688.49089 | 4.84 | PE(32:2) | PE(32:2) | M+H | PE | 14:0/18:2 | 14:00 | 18:02 | NA | 688.4912 |
| 718.53856 | 7.73 | PE(34:1) | PE(34:1) | M+H | PE | 16:0/18:1 | 16:00 | 18:01 | NA | 718.5381 |
| 738.50487 | 6.04 | PE(34:2) | PE(34:2) | M+Na | PE | 16:1/18:1 | 16:01 | 18:01 | NA | 738.5044 |
| 744.55434 | 7.8 | PE(36:2) | PE(36:2) | M+H | PE | 18:1/18:1 | 18:01 | 18:01 | NA | 744.5538 |
| 781.48782 | 3.97 | PI(18:0/12:0) | PI(30:0) | M-H | PI | 18:0/12:0 | 18:00 | 12:00 | NA | 781.4873 |
| 809.52029 | 5.02 | PI(16:0/16:0) | PI(32:0) | M-H | PI | 16:0/16:0 | 16:00 | 16:00 | NA | 809.5186 |
| 807.50578 | 4.17 | PI(16:0/16:1) | PI(32:1) | M-H | PI | 16:0/16:1 | 16:00 | 16:01 | NA | 807.5029 |
| 821.52157 | 4.6 | PI(15:0/18:1) | PI(33:1) | M-H | PI | 15:0/18:1 | 15:00 | 18:01 | NA | 821.5186 |
| 819.50569 | 3.85 | PI(15:0/18:2) | PI(33:2) | M-H | PI | 15:0/18:2 | 15:00 | 18:02 | NA | 819.5029 |
| 835.53687 | 5.27 | PI(16:0/18:1) | PI(34:1) | M-H | PI | 16:0/18:1 | 16:00 | 18:01 | NA | 835.5342 |
| 833.52102 | 4.36 | PI(16:0/18:2) | PI(34:2) | M-H | PI | 16:0/18:2 | 16:00 | 18:02 | NA | 833.5186 |
| 863.56765 | 6.75 | PI(18:0/18:1) | PI(36:1) | M-H | PI | 18:0/18:1 | 18:00 | 18:01 | NA | 863.5655 |
| 861.55394 | 5.41 | PI(18:1/18:1) | PI(36:2) | M-H | PI | 18:1/18:1 | 18:01 | 18:01 | NA | 861.5499 |
| 859.53816 | 4.49 | PI(18:1/18:2) | PI(36:3) | M-H | PI | 18:1/18:2 | 18:01 | 18:02 | NA | 859.5342 |
| 857.52227 | 3.77 | PI(18:2/18:2) | PI(36:4) | M-H | PI | 18:2/18:2 | 18:02 | 18:02 | NA | 857.5186 |
| 809.51787 | 4.28 | PI(32:1) | PI(32:1) | M+H | PI | 16:0/16:1 | 16:00 | 16:01 | NA | 809.5175 |
| 807.50211 | 3.5 | PI(32:2) | PI(32:2) | M+H | PI | 16:1/16:1 | 16:01 | 16:01 | NA | 807.5018 |
| 837.54851 | 5.41 | PI(34:1) | PI(34:1) | M+H | PI | 16:0/18:1 | 16:00 | 18:01 | NA | 837.5488 |
| 907.43497 | 8.62 | PIP(32:3) | PIP(32:3) | M+Na | PIP | 32:03:00 | 32:03:00 | NA | NA | 907.4344 |
| 1185.73835 | 7.96 | PIP(54:2) | PIP(54:2) | M-H | PIP | 54:02:00 | 54:02:00 | NA | NA | 1185.735 |
| 730.46843 | 3.62 | PS(16:1/16:1) | PS(32:2) | M-H | PS | 16:1/16:1 | 16:01 | 16:01 | NA | 730.4665 |
| 760.51579 | 5.52 | PS(16:0/18:1) | PS(34:1) | M-H | PS | 16:0/18:1 | 16:00 | 18:01 | NA | 760.5134 |
| 758.50118 | 4.47 | PS(16:1/18:1) | PS(34:2) | M-H | PS | 16:1/18:1 | 16:01 | 18:01 | NA | 758.4978 |
| 782.49888 | 6.13 | PS(18:1/18:1) | PS(36:2) | M-H | PS | 18:1/18:1 | 18:01 | 18:01 | NA | 782.4978 |
| 732.48125 | 3.67 | PS(32:2) | PS(32:2) | M+H | PS | 16:1/16:1 | 16:01 | 16:01 | NA | 732.481 |
| 760.51199 | 4.59 | PS(34:2) | PS(34:2) | M+H | PS | 16:1/18:1 | 16:01 | 18:01 | NA | 760.5123 |
| 774.53073 | 4.49 | PS(35:1) | PS(35:1) | M-H | PS | 35:01:00 | 35:01:00 | NA | NA | 774.5291 |
| 694.65006 | 10.99 | SiE(18:2) | SiE(18:2) | M+NH4 | SiE | 18:02 | 18:02 | NA | NA | 694.6497 |
| 675.60721 | 9.58 | SiE(18:1) | SiE(18:1) | M+H | SiE | 18:01 | 18:01 | NA | NA | 675.6075 |



**FIGURE S1.** TEM images of nano-Fe_3_O_4_@PCN.


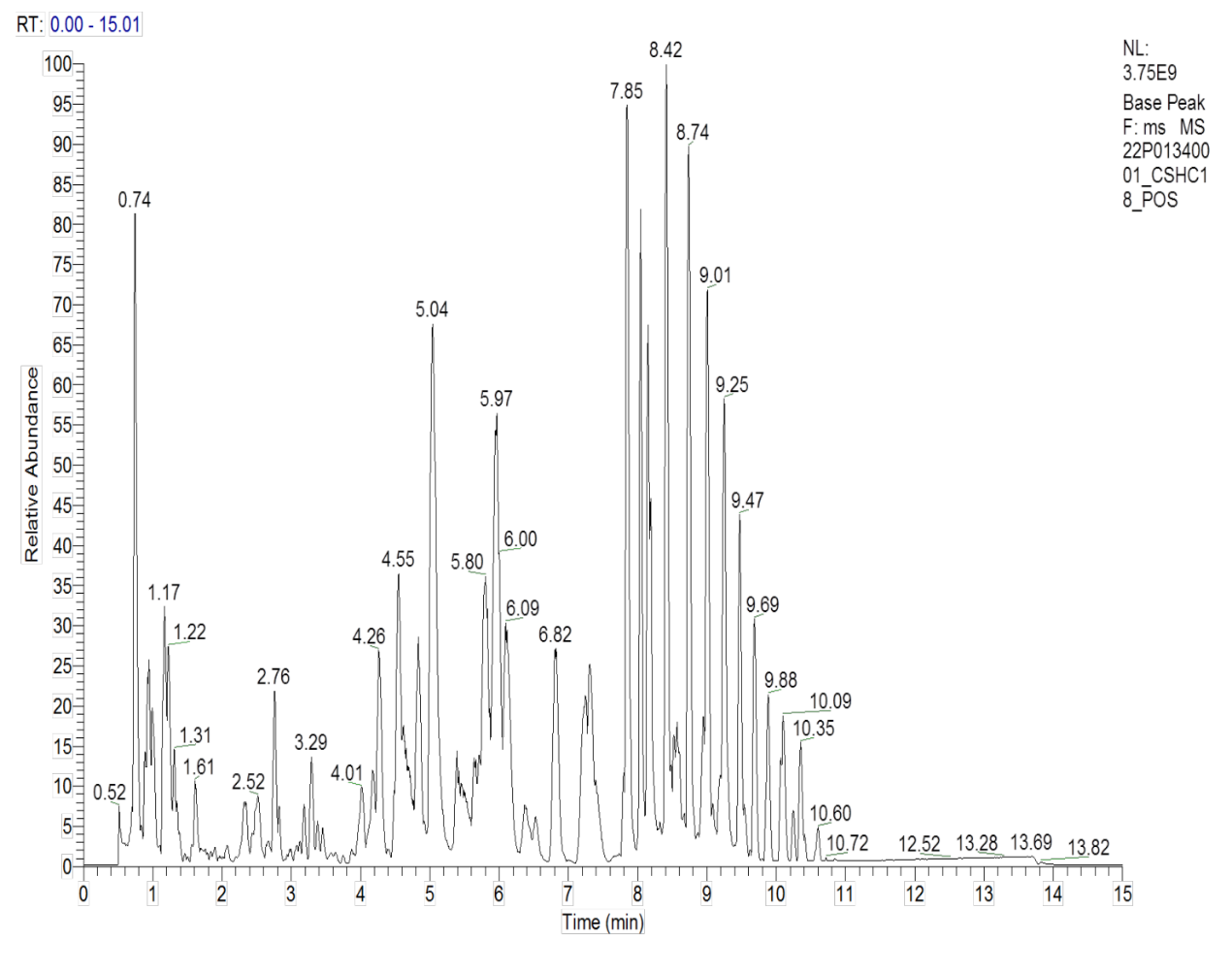


**FIGURE S2.** Typical total ion chromatogram of *S.cerevisiae* cytomembrane lipids extract.


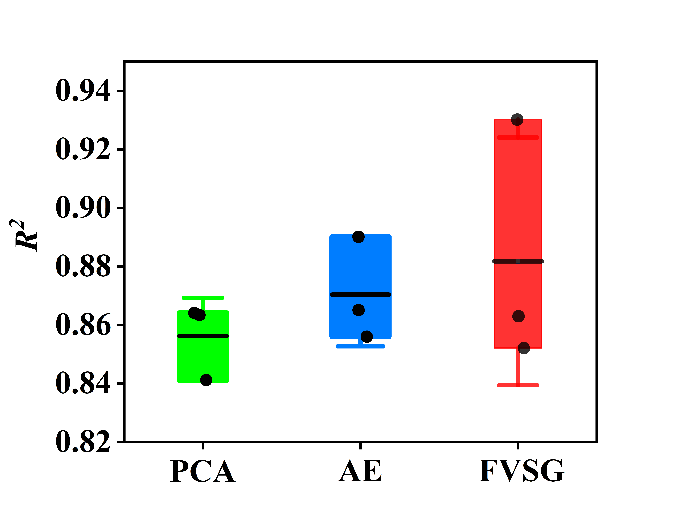
**FIGURE S3.** Test dataset *R^2^* of membrane lipid oxidation and dissipation of PMF.
